# Cholesterol homeostasis: a key to prevent or slow down neurodegeneration

**DOI:** 10.3389/fphys.2012.00486

**Published:** 2013-01-04

**Authors:** Laura Anchisi, Sandra Dessì, Alessandra Pani, Antonella Mandas

**Affiliations:** ^1^Child Neuropsychiatry Unit, Azienda Sanitaria Locale (ASL) n°5Oristano, Italy; ^2^Department of Clinical and Experimental Medicine and Pharmacology, University of MessinaMessina, Italy; ^3^Department of Biomedical Sciences, University of CagliariMonserrato, Cagliari, Italy; ^4^Department of Medicine Sciences, University of CagliariMonserrato, Cagliari, Italy

**Keywords:** neurodegenerative disorders, neuronal membranes, cholesterol homeostasis, cholesterol esterification, drug targets

## Abstract

Neurodegeneration, a common feature for many brain disorders, has severe consequences on the mental and physical health of an individual. Typically human neurodegenerative diseases are devastating illnesses that predominantly affect elderly people, progress slowly, and lead to disability and premature death; however they may occur at all ages. Despite extensive research and investments, current therapeutic interventions against these disorders treat solely the symptoms. Therefore, since the underlying mechanisms of damage to neurons are similar, in spite of etiology and background heterogeneous, it will be of interest to identify possible trigger point of neurodegeneration enabling development of drugs and/or prevention strategies that target many disorders simultaneously. Among the factors that have been identified so far to cause neurodegeneration, failures in cholesterol homeostasis are indubitably the best investigated. The aim of this review is to critically discuss some of the main results reported in the recent years in this field mainly focusing on the mechanisms that, by recovering perturbations of cholesterol homeostasis in neuronal cells, may correct clinically relevant features occurring in different neurodegenerative disorders and, in this regard, also debate the current potential therapeutic interventions.

## Introduction: neurodegeneration and cholesterol

Progressive loss of structure or function of neurons, neurodegeneration, (Bartzokis, 2004) is a common feature for many neurodegenerative diseases such as Alzheimer's disease (AD) (Pani et al., 2009a,b), Parkinson's disease (PD) (Asher, 2011), multiple sclerosis (MS) (Luessi et al., 2012), amyotrophic lateral sclerosis (ALS) (Pratt et al., 2012) and prion disorders (PrD) (Pani et al., 2009a,b). Neurodegeneration may also occur in response to stroke, and head and spinal cord trauma (Thompson, 2008). Typically these diseases affect older people, however, also autistic spectrum disorders (ASD), which includes autism, attention deficit disorder (ADD), attention deficit hyperactivity disorder (ADHD), Asperger syndrome and other disorders that affect children, are thought to be caused by abnormalities in brain function or structure of probable genetic origin (Johnson et al., 2007). Although major advances have been made in the understanding neurodegeneration, its pathophysiology is not yet fully understood and treatment that can prevent or slow down neurodegenerative disorders is still lacking. Therefore, there is an urgent need to identify possible triggers of neurodegeneration in order to develop effective treatment and/or prevention strategies that could ameliorate many diseases simultaneously. Among the factors that until now have been identified as affecting the neurodegeneration development, failures in cholesterol homeostasis are indubitably the most well-known (Anstey et al., 2008; Christie, 2008). Cholesterol is a multifaceted molecule, which serves as essential membrane component, as cofactor for signaling molecules and as precursor for steroid hormones. In the central nervous system (CNS), it is required for brain growth and myelination of axons in the developing brain and for continued axon growth and synapse remodeling in the mature brain (Koudinov and Koudinova, 2003; Dietschy, 2009). Cholesterol in the CNS has also an important role in promoting phase separation within neuron membranes leading to laterally organized regions called lipid rafts (Brown and London, 1998). These regions are enriched in unesterified (free) cholesterol (FC) and other specific lipid compounds (sphingolipids, plasmenylethanolamine, anionic phospholipids, plasmalogens, and arachidonic acid containing phospholipids) (Simons and Ehehalt, 2002; Korade and Kenworthy, 2008). Lipid rafts allow a lateral separation of the membrane leading to functionally distinct regions of the membrane. Some lipid rafts are needed in order to export proteins out of the cell, others are used to anchor specific proteins in the membrane and keep protein clusters together. Thus, they are protein and lipid fluctuating microdomains important for protein trafficking and sorting, for cell signaling, and for manifold cellular processes. Reduction of cellular cholesterol leads to disruption of raft functions (Bari et al., 2005; Murai, 2012). Finally, it is also important to mention the role of cholesterol in the synthesis of neurosteroids. These compounds act as allosteric modulators of neurotransmitter receptors and are synthesized, especially in myelinating glial cells, from cholesterol or steroidal precursors (Mellon and Griffin, 2002; Kimoto et al., 2006). On the basis of the above information, it can deduce that, changes into one or more of the integrated sets of homeostatic mechanisms that finely regulate cholesterol content in neurons, could adversely affect the normal brain functions leading to neurodegeneration; a better understanding of several molecular mechanisms of cholesterol involvement in this process might thus represent a useful tool to identify biomarkers for neurologic disorders as well as targets for pharmacological interventions. Here we review some of the main results on the subject area reported in recent years with the aim to provide plausible explanations of how alterations in brain and blood lipid metabolism contribute to neurodegeneration. A major focus will be on the mechanisms that, by restoring cholesterol homeostasis perturbations in neuronal cells, may improve clinically relevant features found to be present in different major neurodegenerative diseases.

## Cholesterol in neuronal plasma membrane

The plasma membrane (PM) consists of both lipids and proteins, its fundamental structure being the phospholipid bilayer, which forms a stable barrier between two aqueous compartments inside and the outside of the cells. Cholesterol is a ubiquitous component of all PMs, including those of neurons, where it determines their permeability, fluidity, and mechanical properties. In the PMs, cholesterol in its free form has the tendency to form regularly distributed lateral structures giving rise to the formation of highly ordered nano-scale membrane domains called lipid rafts (Simons and Ehehalt, 2002; Korade and Kenworthy, 2008) which have important roles in numerous cellular functions. Despite many studies, however, how cholesterol is unique in promoting the formation of lipid rafts and what emerges from the interactions of it with other lipids still remain unresolved questions. It has shown that lipid rafts generally contain 3–5-fold the amount of cholesterol found in the surrounding bilayer (Pike, 2003); this cholesterol enrichment seems to be necessary to hold rafts together and to modulate raft-dependent cell functions, having sterols extraordinary ordering capacity. In neuronal PMs, FC, due to its structure and the saturation of the hydrocarbon chains, preferentially interacts with phosphatidylcholines and sphingolipids such as sphingomyelin; its small head-group and the rigid ring structure prevent almost all direct sterol-sterol contacts helping cholesterol to avoid the exposure of hydrophobic membrane regions to water which would give rise to a very unfavorable contribution to the free energy. The cholesterol biosynthetic pathway shows a systematic removal of methyl groups from the steroid ring. Each removal step optimizes sterol properties in terms of ordering and condensing effects (Aittoniemi et al., 2006). Analysis of the molecular orientations have revealed that cholesterol, especially at higher concentrations, clearly promotes opposite configurations resulting in a sandwiching-like pattern—two cholesterol molecules sandwich a phospholipid. This configuration is found to be more likely for saturated than unsaturated lipids in agreement with cholesterol's higher tendency to order and condense saturated than unsaturated lipids, and this may at least in part explain why the liquid-ordered phase is not observed at small cholesterol concentrations (Martinez-Seara et al., 2010). The above discussed organization of cholesterol highlights the importance of cholesterol's unique molecular structure in promoting the formation of the liquid-ordered phase. In this spirit it seems obvious that cholesterol molecules do not function alone but they do so in a co-operative manner, highlighting the importance of understanding collective ordering phenomena in PMs. All these structural and functional roles of cholesterol in PMs, imply that even minor changes in neuronal PM cholesterol concentrations can lead to neurodegeneration.

## Free cholesterol in the central nervous system

Human CNS contains over 23% of the total body cholesterol while accounting for only 2% of the total body mass. Because the brain's cholesterol metabolism is almost exclusively segregated from the peripheral circulation by the blood brain barrier (BBB), cholesterol metabolism in brain is different from that in periphery. In the adult brain it is mainly in the FC form and only trace amount of esterified cholesterol (CE) are present. Nearly 95% of this FC is synthesized de novo from acetate, predominantly in the endoplasmic reticulum (ER) of glial cells—astrocytes and, to a lesser extent, oligodendrocytes—with only small amounts synthesized in neurons (Dietschy and Turley, 2004; Dietschy, 2009). FC pool in adult human brain (about 490 mg/kg) is distributed among three different compartments: (1) myelin membranes (2) neuron PMs and (3) glial cells. Myelin membranes account for 70–80% of FC (about 380 mg/kg), whereas the remaining 110 mg/kg presumably resides in the membranes of cellular elements: about 11 mg/kg in neurons (~10% of brain cells), and about 99 mg/kg in different types of glial cells and vascular elements (Jiménez-López et al., 2010). The specificity of lipids, mainly FC and their patterns of accumulation during development (Dietschy and Turley, 2004; Dietschy, 2009), contributes to the direct and permissive roles that these compounds play in signal transduction as well as in both activation and deactivation of raft resident proteins involved in multiple brain functions (George and Wu, 2012). The human brain increases dramatically in size and complexity in the first year of life (Blüml et al., 2012), and synthesis of lipid components increase in proportion to the progressive increase of brain volume (Dietschy and Turley, 2004). From birth to adolescence, there is a 4-fold increase in the volume of the human brain; this increase however is not uniform: there is differential growth between subcortical and cortical regions, and between different regions of cortex. Whereas there is a rapid increase in synaptogenesis around the time of birth for all cortical areas studied, the most rapid burst of synapse formation and the peak density of synapses occur at different ages in different areas (Johnson, 2001). For example, prefrontal cortex is one of the last regions of the brain to reach maturation, in this region of brain synaptogenesis starts between 3 and 4 months, and the density of synapses increases slowly and does not reach its peak until after the first year. The prefrontal cortex is the part of the frontal lobes lying just behind the forehead and is responsible for cognitive analysis and abstract thought, and the moderation of “correct” behavior in social situations. Although some controversy, the consensus is that brain structures have the overall appearance of those in the adult by 2 years of age, and that all the main white matter fiber tracts can be observed by 3 years of age (Matsuzawa et al., 2001; Paus et al., 2001). In some reports, it is suggested that after a rapid increase in grey matter (predominantly composed of neuron cell bodies and unmyelinated axons), brain volume up to ~4 years of age, there is then a prolonged period of slight decline that extends into adult years (Johnson, 2001). A role for loss of white matter in neurodegeneration has been previously reported (Bartzokis, 2004), however, whether the decline in brain volume is associated with loss of white matter remains unknown (Matsuzawa et al., 2001). It is important to remember the white matter is so called because the high concentration of myelin which is constituted of about 70–85% of lipids, mainly cerebroside, cholesterol, and phosphatidylcholine. As cholesterol is an essential component of myelin in white matter, not surprisingly, the size of the sterol pool in the CNS increases (or decreases) in a proportional manner to white matter volume (Dietschy and Turley, 2004). Humans synthesize high rates of cholesterol after birth mainly in oligodendrocytes, as the CNS matures and myelin production decreases to very low levels, CNS cholesterol pool reaches a constant value. In summary, the majority of brain cholesterol is accumulated between neonatal period and adolescence when neurons become surrounded by specialized PMs termed myelin (Dietschy and Turley, 2004). After myelination, the metabolism of cholesterol in the adult brain is characterized by a very low turnover and minimal losses (Morell and Jurevics, 1996). However, recent results indicate that both cholesterol synthesis and degradation are active in the adult brain as well and that alterations in these mechanisms profoundly influence higher-order brain functions (Martin et al., 2010; Orth and Bellosta, 2012).

## CE and esterification in the CNS

As already reported, cholesterol, in adult brain, is mainly in the free form, while only trace amounts of CE are present. However, a significant portion (up to 5%) of total cholesterol is found to be esterified in the developing brain. It follows that the CE concentration in the brain falls between very early stage of development and late stages of adulthood (Husuf, 1992). Interestingly, a fast and temporary rise in the concentration of CE occurs at a time that corresponds to the onset of myelinogenesis. This transient increase appears to be a universal phenomenon, because it has been found in all mammalian brains that have been studied so far. In particular, (Yusuf et al., 1981) reported that in humans the CE concentrations in the three main parts of brain, forebrain, cerebellum, and brainstem, were highest at 13–15 weeks and rapidly fell during growth. Transient rises in CE concentrations were also seen around birth in the forebrain and few months after birth in the cerebellum, a period coinciding with the onset of active myelination in the respective brain areas. Thus, the increase in the CE in the developing human forebrain appears to be governed by two events, one representing brain growth and the other representing the active phase of myelination, supporting the idea of a close association between CE and the process of myelination.

Beside during physiologic development, changes in CE metabolism have been reported in many different neurologic disorders including AD, ALS, MS, and PrD. In particular, an increase in CE concentration has been found in AD (Puglielli et al., 2001), MS (Machtoub et al., 2011) and PrD brains (Pani et al., 2011) and in spinal cords of ALS patients (Cutler et al., 2002). In animal models it has been found that brain CE accumulation and increased activity of the esterifying enzyme, acyl coenzyme A (CoA): cholesterol acyltransferase (ACAT1) precede the clinical phenotype (Posse de Chaves and Narayanaswami, 2008); in addition, exposure of cultured hippocampal neurons to neurotoxic components (i.e., amyloid β-peptide, Aβ) and of cultured motor neurons to oxidative insults, produces a discrete accumulation of intracellular CE (Cutler et al., 2002). Worthy of mention, stearoyl-CoA desaturase (SCD), an ER enzyme that catalyzes the biosynthesis of monounsaturated fatty acids (MUFAs) from saturated fatty acids has been recently shown to have a role in providing optimal substrate for cholesterol esterification (Paton and Ntambi, 2009). This is particularly important in light of the recent study by Astarita et al. (2011) that suggests that cholesterol/CE homeostasis (SCD-ACAT) is altered in AD. Although it is still unclear if elevated levels in CE are the result of cell death process or if they directly contribute to neurogegeneration, abnormalities in CE metabolism appear to be another characteristic of neurodegenerative diseases.

## FC and neurodegeneration

**Figure 1:**
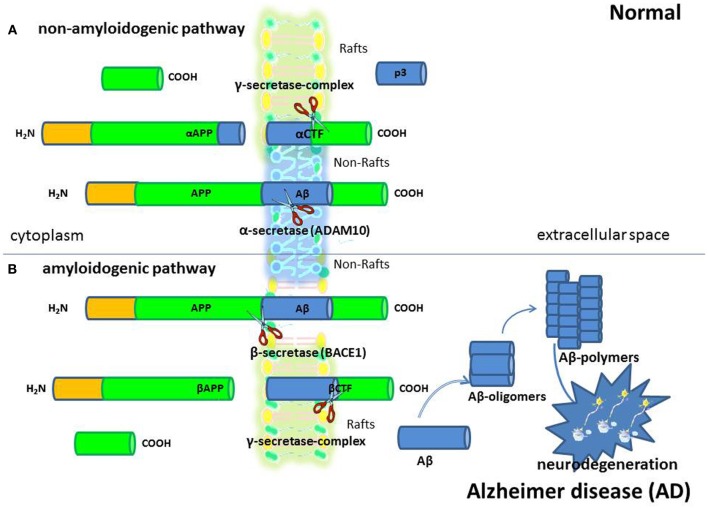
**Alzheimer's disease (AD).** AD is a progressive neurologic disease of the brain leading to the irreversible loss of neurons and the loss of intellectual abilities, including memory, and reasoning, which become severe enough to impede social or occupational functioning. Although scientists are still trying to fully understand the cause/s of AD, the formation of amyloid β (Aβ) protein positive neuritic plaques is considered one of the most important characteristic of AD so much so that Aβ deposition in the brain is used to diagnose the disease in autopsy. Aβ peptides are fragments from a larger protein called amyloid precursor protein (APP), a transmembrane protein that penetrates through the neuron's membrane. They are generated in the amyloidogenic pathway **(B)** of APP processing by sequential proteolysis by β- (BACE1) and γ-secretases. In the alternative non-amyloidogenic APP processing pathway **(A)**, α-secretase cleaves within the Aβ peptide region and prevents Aβ generation.

**Figure 2:**
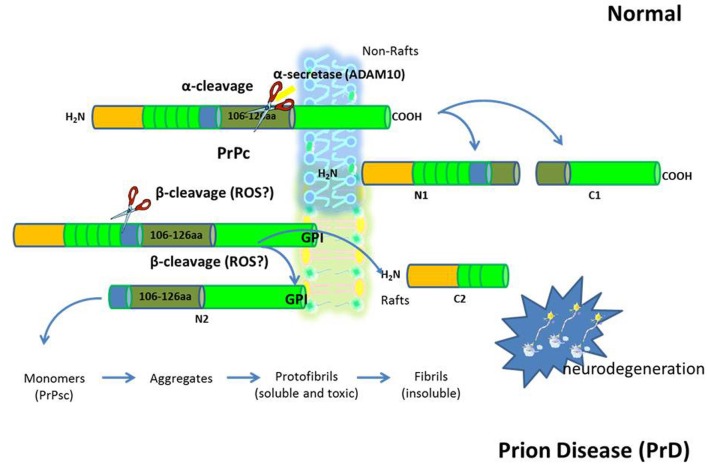
**The prion diseases (PrD).** PrD are a large group of related neurodegenerative conditions, which affect both animals and humans. Included are Creutzfeldt–Jakob disease and Gerstmann-Strãussler-Scheinker in humans, bovine spongiform encephalopathy, or “mad cow disease” in cattle, chronic wasting disease in mule deer and elk, and scrapie in sheep. Prion diseases are unique in that they can be inherited, can occur sporadically, or can be infectious. The infectious agent in the PrD is composed mainly or entirely of an abnormal conformation of a host-encoded glycoprotein called the prion protein (PrPc). The replication of prions implies the transformation of the normal version of this protein PrPc into a misfolded form (PrPsc). An increased concentration of PrPc at the membrane leads to a structural transition toward abnormal intermolecular beta sheet. This dimerization could well be the initial step on the pathway of the conversion into PrPSc.

Growing evidence indicates that membrane lipid rafts are involved in the generation of Aβ and PrPsc amyloidogenic-peptides from amyloid precursor protein (APP) and cellular prion protein (PrPc), (deposition of which in brain parenchyma and vessel walls are the major pathological feature of AD and PrD, respectively), possibly through the creation of a favorable lipid environment (Figures 1 and 2) (Pani et al., 2011). It has been reported that, when APP and PrPc molecules occupy a lipid raft region of the membrane, they are more accessible to and, thus, preferentially cleaved by amyloidogenic enzymes (i.e., raft resident β-secretase or BACE1). On the other hand, when APP and PrPc molecules are outside rafts, they appear to be preferentially cleaved by non-amyloidogenic non-raft resident α-secretase (ADAM 10) (Simons and Ikonen, 1997). It has been thus suggested that APP and PrPc in the brain are present in two cellular pools: one, outside rafts, where these two proteins are processed in a normal way; the other, inside the rafts, where abnormal cleavage takes place (Simons and Ikonen, 1997). If it is so, it can be assumed that compartmentalization of membrane proteins depends by spatial distribution of the lipid raft microdomains which in turn is regulated by the levels of FC and therefore that maintenance of cholesterol homeostasis is essential for neurons. If cholesterol levels are higher or lower than the physiological range; several metabolic pathways of compensation can be activated that if continued for long periods can lead to neurodegeneration through different mechanisms.

## CE and neurodegeneration

A significant amount of genetic, biochemical, and pharmacological data highlighted that modifications in cholesterol esterification and trafficking are associated with Aβ and PrPsc biogenesis (Puglielli et al., 2001; Hutter-Paier et al., 2004; Bhattacharyya and Kovacs, 2010; Huttunen et al., 2010; Pani et al., 2007a,b,c, 2011; Orrù et al., 2010a,b,c). Moreover, several studies in various cell and animal models of AD evidenced that, genetic or pharmacological inhibition of ACAT activity markedly suppresses Aβ generation. Inhibition, by RNAi-induced decrease of ACAT expression, or by ACAT inhibitors (i.e., CP-113,818 and avasimibe), of cholesterol esterification prevents Aβ generation and its deposition in cell cultures, and markedly reduced neuritic plaques in AD murine models. More recently, Bryleva et al. (2010) by using a combined mouse genetic and biochemical approach, showed that ablation of ACAT1 gene in triple transgenic (3XTg-AD) mice leaded to more than 60% reduction in full-length human APP and its proteolytic fragments, and to an improvement in cognitive deficit. Microarray studies during disease progression of different mouse strain-prion strain combinations revealed that ACAT1 expression gene was activated as early as 10 weeks post prion infection. At advanced stages of the disease (18–22 weeks after infection), only the ACAT-encoding gene—out of all the genes involved in cholesterol synthesis, trafficking, and processing examined—was constantly up-regulated (Hwang et al., 2009). Changes in cholesterol esterification similar to that observed in insulted brains were observed by us in peripheral cells. In particular, increased CE levels were found in skin fibroblasts and peripheral blood mononuclear cells (PBMCs) from AD patients and from sheep with natural scrapie (Pani et al., 2007a,b,c, 2009a,b). It has been shown that about 85% of AD patients displayed significantly higher PBMCs-CE levels than cognitively normal age-matched controls. Of note is that parallel analysis in AD first-grade relatives revealed that up to 30% of them exhibited a peripheral cholesterol pattern similar to that of AD patients. Interestingly, higher CE pool was also observed in skin fibroblasts of healthy sheep with a scrapie susceptible prion genotype compared to sheep carrying a scrapie-resistant prion genotype (Pani et al., 2007a). In agreement with findings of early ACAT1 activation in mouse brains of experimental scrapie (Hwang et al., 2009), and in brains of AD (Pani et al., 2011), we also found increased ACAT1 expression in both skin fibroblasts and PBMCs from AD patients (Pani et al., 2009a,b) and in skin fibroblasts from scrapie-affected and scrapie-susceptible sheep (Pani et al., 2007a).

## Evidence for altered lipid homeostasis in autistic spectrum disorders (ASD)

Nowadays, more and more researchers talk about ASD as neurodegenerative disorders (Hagerman, 2006). This is based on the identification in some autistic children of pathophysiological features such as on-going systemic and CNS redox abnormalities and inflammation, and the evidence of brain volume and neuron loss. Genetic alterations, prenatal exposure to viruses and/or toxins maternal and fetal immune interactions (Takahashi et al., 2001; Nicolson et al., 2007), and other as yet undetermined causes may contribute independently or synergistically to the development of autism. Recently, evidence has been reported supporting a role for cholesterol also in the pathogenesis of these disorders. Three mechanisms working in concert have been hypothesized to explain how low cholesterol levels may contribute to sporadic ASD (Lee and Tierney, 2011): (1) impaired sonic hedgehog (SHH) signaling molecules which are involved in the regulation of organogenesis including the organization of the brain. During embryonic development, SHH is covalently modified with both palmitate and cholesterol and secreted as part of a lipoprotein complex that regulates brain morphogenesis through the patched/smoothened signaling system (2) alterations in membrane lipid raft structure and protein function resulting in abnormal synaptic plasticity, and (3) impaired neurosteroid synthesis. Starting from these considerations, and from the notion that many scientists consider AD “nothing more than autism in the elderly,” we thought that it might be interesting to determine CE levels in PBMCs from children with sporadic autism. In addition, since, many studies have outlined the dimensionality of autism in regard to its comorbidity with other neurodevelopmental disorders such as ADHD we also determined CE levels in PBMCs from children affected by ADHD. This is the most common psychiatric disorder in children, affecting about 3–5% of children globally (Spencer et al., 1999) and is characterized primarily by the co-existence of attention problems and hyperactivity, with symptoms starting before seven years of age (Waschbusch et al., 2002; Wilens et al., 2002). In about 30–50% of those individuals diagnosed in childhood, symptoms persist into adulthood (Kuhne et al., 1997). In a manner similar to that observed in the AD patients, both autistic and ADHD children unveiled higher CEs levels in cytoplasm of their PBMCs compared to that of age-matched control children (Anchisi et al., 2013). These results lead us to hypothesize that increased intracellular CE levels in PBMCs might represent a peripheral preclinical and clinical marker of neurodegeneration both in old and young affected subjects. The above-mentioned observations leave little doubt that cholesterol esterification is involved in neurodegeneration and suggest that CEs and ACAT, beside biomarkers, might become important therapeutic targets in treating several neurodegenerative disorders. Thus the last part of the review is aimed at providing information on the research in the field, which may help to understand the molecular mechanism/s underlying the link between changes in cholesterol esterification and neurodegenerative processes.

## Evidence that low and not high neuronal membrane cholesterol levels contribute to neurodegeneration

In spite of the intense research carried out in recent times, the role of cholesterol as a risk factor for neurodegeneration remains controversial and it is still debated if high or low neuronal membrane cholesterol levels are involved in neurodegenerative disorders. In AD, the high cholesterol model assumes that higher FC levels in neuronal membrane may exert harmful effects (Simons et al., 1998) and implies that lowering CNS cholesterol would be beneficial to neuronal function. Indeed, there is a large body of evidence suggesting that increased levels of PM cholesterol promote the amyloidogenic processing of APP and thereby contribute to the key series of molecular proteolytic events widely believed to underpin the etiology of AD. A number of genes involved in cholesterol homeostasis have been identified as susceptibility loci for sporadic or late-onset AD and cholesterol and other specific lipids have been also shown to enhance the propensity of Aβ to form neurotoxic aggregates (Beel et al., 2010). It has been proposed that high membrane cholesterol by increasing co-clustering of lipid rafts induces co-localization of APP-BACE1 (Simons et al., 1998; Wolozin et al., 2000); this in turn may influence APP processing as well as its location cleavage stimulating the amyloidogenic pathway that leads to increased Aβ production. On the contrary, the low membrane cholesterol model envisages a protective role of relatively high amounts of membrane cholesterol assuming that APP is located in non-raft membrane domains (Ledesma and Dotti, 2006, 2012). High membrane cholesterol would maintain APP and BACE1 separated into different membrane domains thus reducing Aβ generation (Ledesma and Dotti, 2006, 2012; Pani et al., 2011). The idea that high cholesterol levels might be responsible for neurodegeneration was mainly supported by findings that cultured human hippocampal neurons and neuroblastoma cells expressing the amyloidogenic Swedish mutation of APP, treated with statins, the well-known inhibitors of cholesterol synthesis, exhibited reduced Aβ production and inflammatory response to Aβ aggregates and increased α-secretase activity (Höglund et al., 2006). Statin treatment also contributed to maintain cholesterol distribution in the cell membrane and normal function of membrane proteins while delaying APP cleavage and Aβ production (Höglund et al., 2006). Decreased Aβ production has also been shown in guinea pigs treated with high doses of statins (Ledesma and Dotti, 2005). However, several new data raise doubts on the beneficial effects of statins in neurodegeneration and on the notion that high cholesterol in neuronal membranes is associated with brain dysfunction. This includes the finding of the increase of amyloid production and presence of neurodegeneration in female mice treated with statins able to cross BBB, the evidence that defective cholesterol metabolism and trafficking are present in the familial forms of a number of neurodegenerative diseases and the observation that the hippocampus of certain AD patients present a moderate, yet significant, reduction in membrane cholesterol (Abad-Rodriguez et al., 2004). As pointed out above, in neurons, cholesterol in mainly located at the PMs and membrane environment is of fundamental importance in regulating membrane protein degradation and in modulating protein/peptide fibrillization. Actually, cholesterol is crucial for the myelin as well as for the synapse. Mutant mice lacking the ability to synthesize cholesterol had severely disturbed myelin in their brains, and exhibited ataxia (un-coordinated muscle movements) and tremor indicating that cholesterol is an indispensable component of myelin membranes that contribute proper production of the myelin sheath and their function in synapses (Saher et al., 2005). All these findings call for a reconsideration of the high cholesterol model, and suggest that low rather than high levels of cholesterol in neuronal membranes could be detrimental for cells and might also promote neuronal degeneration. However, it has been demonstrated that for model cell lines the overall cholesterol content can change from 2 to 4-fold at different phases of cell growth (Cansell et al., 1997; Takahashi et al., 2007). Thus, a full understanding of the *in vivo* dynamic variability of cholesterol and lipid raft content in plasma or organelle membranes as a function of cellular physiological state is still lacking.

## Molecular mechanisms linking cholesterol esterification to neurodegeneration

**Figure 3:**
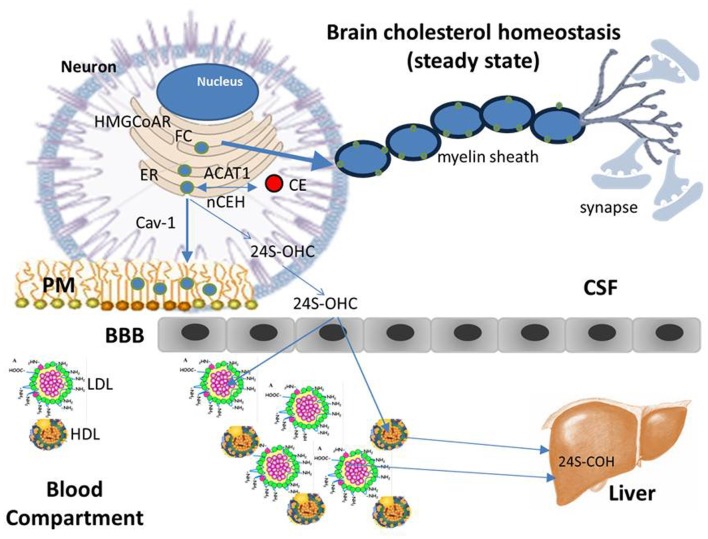
**Cholesterol homeostasis (steady state).** Differently from the most tissues that take up cholesterol from circulating plasma lipoproteins through the classic mechanism of the low density lipoprotein receptor (LDLR), due to the BBB, brain does not have direct access to cholesterol carried by plasma lipoproteins and therefore, it meets its cholesterol needs through de novo synthesis mainly in glial cells, with only a small amount of cholesterol synthesized in neurons. Glial cells package neo-synthesized cholesterol into Apo E—containing lipoprotein particles, which in turn are secreted into the CSF through the ATP-binding cassette transporter 1 (ABCA1). Apo E-containing lipoproteins are then taken up by neurons and FC released is transported for subsequent metabolism and trafficking to other intracellular sites. Neurons keep constant their cholesterol concentrations through the same homeostatic mechanisms regulating the intracellular cholesterol metabolism in peripheral tissues: cholesterol synthesized in the ER, as well as that released by Apo E—containing lipoprotein catabolism, moves to PMs, in part, by interacting with Cav-1. Once the capacity of PMs and other compartments to absorb cholesterol is exceeded, cholesterol is transported back to the ER, where, in a small part it is esterified by ACAT and accumulated as lipid droplets. The large part of excess cholesterol, however, is converted into 24S-OHC, crosses the BBB, enters the plasma, and is delivered to the liver for excretion into bile.

**Figure 4:**
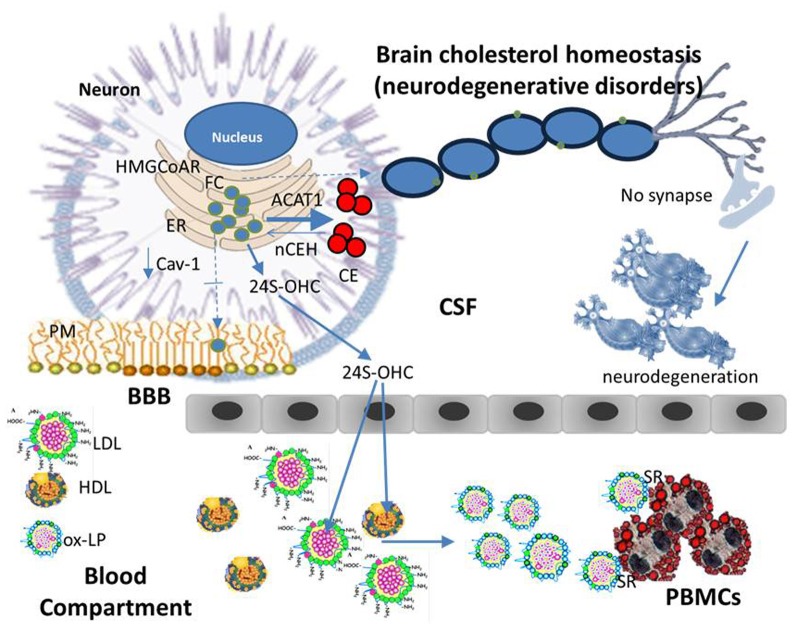
**Cholesterol homeostasis (neurodegenerative disorders).** An imbalance of one or more of finely regulated homeostatic mechanisms that lead to even modest changes in ER-FC pool in neurons can cause a serious and sometimes fatal neurologic disorder. Namely, if a reduction in the transport of cholesterol between ER and PM occurs as a consequence of genetic and/or environmental factors, neuronal ER-FC increases. This increase may cause the activation of ACAT1 leading to an increased CE synthesis while reducing the distribution of FC in raft-domains. If these alterations persist over time the consequences may be: rafts disassembly, demyelination, alterations in synapse formation and function, in other words, neurodegeneration. Beside ACAT1 activation, an increase in ER cholesterol pool of neurons may also activate the CYP46A1 thereby enhancing the levels of circulating 24S-OHC. This is an oxygenated derivative of cholesterol able to cause lipoprotein oxidation (ox-LP). With a mechanism similar to that described for atherosclerosis, ox-Lp might be recognized by scavenger receptors (SR) on the surface of white blood cells, which in turn may be engorged with CE and become foam cells.

Taking into account the above and data obtained in our laboratory, a model describing some mechanisms linking cholesterol esterification to neuronal degeneration could tentatively be proposed (Figures 3 and 4). It is well known that cells and tissues, including brain, are protected from the accumulation of potentially toxic FC excess by ACAT1–mediated esterification and by cholesterol efflux (Tabas, 2002), ACAT activity being allosterically activated by the presence of high FC levels in ER (Chang et al., 2001). Cellular cholesterol undergoes a continuous cycle of esterification and ester hydrolysis; net breakdown of CE taking place when ER–FC levels decline. The enzyme responsible for the degradation of CE is neutral cholesterol ester hydrolase (nCEH). Under physiological conditions intracellular CE levels in brains are very low and generally do not exceed the nCEH capacity to re-hydrolize CE to FC and to recycle FC back to PM (Pani and Dessì, 2003). In neurons, if in excess, a part of ER–FC is converted to CE by ACAT1 located at the ER and stored as cytoplasmic lipid droplets, another part leaves the brain (Dietschy, 2009). FC does not across the BBB, therefore before to exit CNS, it is converted into 24S-hydroxycholesterol (24S-OHC) and in this form moves from neurons via the ATP-binding cassette transporter A1 (ABCA1) pathway, through cerebrospinal fluid (CSF), cross the BBB, and is released into the systemic venous circulation. The fate of the 24S-OHC once it reaches the circulation has not yet been defined. An accurate method based on isotope dilution-mass spectrometry showed that in blood compartment 24S-OHC is mainly associated with HDL and LDL (Babiker and Diczfalusy, 1998), suggesting that steady-state plasma 24S-OHC levels follows the metabolic fate of cholesterol in HDL and LDL (i.e., uptake by the liver). Since most of the circulating 24S-OHC arises from brain cholesterol, its levels are considered a measure of cholesterol turnover in the CNS (Orth and Bellosta, 2012). Cells in the CNS synthesize all of their own cholesterol in the ER from acetyl CoA through the mevalonate pathway. The rate-limiting step of the mevalonate pathway is the conversion of hydroxyl-methyl-glutaryl-CoA (HMG-CoA) to mevalonate by HMG-CoA reductase. Both these and several other enzymes that function in later steps of cholesterol synthesis are integral ER membrane proteins. In the ER, FC levels fluctuate much more than that in PMs and are considered the major regulators of the cellular cholesterol homeostatic machinery. Once synthesized, FC leaves the ER, thereby helping to maintain low ER sterol content and is rapidly targeted to PMs where, depending on the type of CNS cells is utilized for membrane turnover and axonal growth or become available for extracellular apoprotein E (Apo E) acceptors (astrocytes) (Dietschy, 2009; Orth and Bellosta, 2012). In summary, the ER, where many critical enzymatic reactions of cholesterol metabolism take place, is relatively cholesterol poor, thus maintenance of cellular cholesterol homeostasis necessitates the transport of cholesterol between subcellular membranes and PMs and eventually its exchange with Apo E and/or ABCA1 for efflux. These findings imply that an imbalance of one or more of these finely regulated homeostatic mechanisms capable of causing even modest changes in ER–FC pool, may contribute to serious and sometimes fatal conditions. In this way, it is plausible to suppose that, if a reduction in the transport of cholesterol between ER and PMs occurs as a consequence of genetic and/or environmental factors, ER–FC in neurons may increase. This increase activates ACAT1 leading to abnormal CE accumulation while membrane cholesterol and its distribution in raft-domains are reduced. If this altered transport persists over time the consequences will be: rafts disassembly, demyelination, alterations in synapsis formation and function (neurodegenerative disorders). Considering that caveolin-1 (Cav-1) is a cholesterol-binding protein that delivers newly synthesized cholesterol at the ER to the PMs (Smart et al., 1996), the fact that embryonic fibroblasts and peritoneal macrophages from Cav-1 null mice were found enriched in CEs but depleted of FC membranes compared with their wild-type counterparts (Chang et al., 2006), supports these conclusions. Signs of premature neuronal aging and degeneration, increased Aβ, decreased cerebrovascular volume and reduction in synapses were also observed in brains of young Cav-1 null mice compared to young WT mice (Head et al., 2010). Very low mRNA levels of Cav-1 and particularly nCEH were found by us in skin fibroblasts and in PBMCs from patients with AD (Pani et al., 2009a,b). Interestingly, neuron-targeted re-expression of Cav-1 in Cav-1 null neurons *in vitro* decreased Aβ expression (Head et al., 2010).

Beside ACAT1 activation, an increase in ER cholesterol pool of neurons may activate the enzyme cholesterol 24-hydroxylase (CYP46A1) thereby enhancing production of 24S-OHC (Shafaati et al., 2007). The oxysterol is then secreted into CSF, and through the BBB, delivered into the circulation. Really, elevated CSF-24S-OHC concentrations have been found in patients with AD compared to age-matched controls (Prasanthi et al., 2009), this elevation was even more pronounced in CSF of moderate cognitive decline—mild or early stage of AD in which an increase of 24S-OHC was also consistently observed in the plasma compartment. In plasma, 24S-OHC is mainly present in HDL and LDL in its esterified form (24S-OHCE). At this point, it should be mentioned that 24S-OHC is an oxygenated derivative of cholesterol and consequently a potential inducer of LDL oxidation. In agreement increased levels of circulating oxidized LDL (ox-LDL) were found in patients with neurologic disorders (Ehnholm, 2009). With a mechanism similar to that described for atherosclerosis, ox-LDL might be recognized by scavenger receptors (SR) on the surface of white blood cells, which in turn may be engorged with CE. Such a scenario may help to explain why subjects with neurological disorders frequently present an accumulation of CE in their PBMCs. According to this model, progressive FC accumulation in ER membranes would intensify ACAT1 dysfunction leading to neurodegeneration. Finally, efflux pathways may also be inhibited by conversion of cholesterol into oxysterols. This information indicates that brain cholesterol alterations during neurodegeneration involve dynamic modifications in cholesterol homeostatic networks, which are reflected in peripheral tissues, therefore, the use of therapeutic strategies, aimed at re-establishing right cholesterol membrane amounts and their differential distribution in lipid rafts, would contribute to successful treatment/prevention of neurodegenerative disorders.

## CE as drug target for prevention and/or treatment of neurodegeneration

In previous studies we have found that cell treatments with combinations of various cholesterol interfering drugs showed synergic anti-prion effects, apparently by restoring cholesterol homeostasis in infected cells (Orrù et al., 2010c) The lysosomotropic drugs quinacrine and chlorpromazine, which, in addition to several other effects (Dohura et al., 2000) respectively determine cell cholesterol redistribution (Klingenstein et al., 2006) and activation of cholesterol biosynthesis (Fernø., 2006), transportation and efflux (Vik-Mo et al., 2009), reduced up to 10-fold their EC50 against PrPsc when associated with a CE inhibitor (Varghese et al., 2005), or with the cholesterol-trafficking modulators such as progesterone or verapamil (Butler et al., 1992; Debry et al., 1997). CE levels directly correlate with Aβ production (Bhattacharyya and Kovacs, 2010), as already mentioned, in fact, experiments in cellular and animal models of AD showed dramatic reduction of Aβ generation and deposition upon inhibition of ACAT. Interestingly, a report on Aβ-positive neurons of autopsied brain tissue from AD patient not only showed the presence of lipid droplets in neurons, but also showed a positive correlation between Aβ levels and lipid droplets (Gomez-Ramos and Asuncion Moran, 2007). Although, at present, it is not possible to form a complete picture of the functionally relevant causal relations, ACAT1 has becoming an excellent potential drug target for neurodegeneration. Inhibition of ACAT1 reduces the intracellular CE and storage, leaves more FC available for the membrane compartment and causes a decrease in Aβ production (Bhattacharyya and Kovacs, 2010).

The knowledge of the critical role played by ACAT in the formation of CE-enriched cells (foam cells), the accumulation of which has an integral part into the development of atheroma, led to the discovery of several inhibitors of this enzyme (Chang et al., 2009). Theoretically, inhibition of ACAT, by blocking the esterification of cholesterol, could prevent the transformation of macrophages into foam cells and slow the progression of atherosclerosis. In some animal models, ACAT inhibitors were remarkably effective in reducing the formation of atheromas (Bocan et al., 1993; Kusunoki et al., 2001), however, no indication of whether this effect was due to a reduction in the number of cells or to the extent of CE enrichment or to both was reported. On the contrary, some studies involving genetically engineered mice have suggested that the inhibition of ACAT1 may promote atherosclerosis (Perrey et al., 2001). In addition, recent clinical trials of ACAT inhibitors in humans failed to show a therapeutic efficacy and this prompted researchers to pronounce the imminent death of ACAT inhibitors as a viable anti-atherosclerotic therapy (Farese, 2006). ACAT inhibitors are currently not marketed and more research is needed to develop safe and effective inhibitors for human use. In 2008, a study used the mammalian target of rapamycin (mTOR) inhibitor, rapamycin, to treat learning disabilities associated with a disease called tuberous sclerosis complex (TSC) in mice (Ehninger et al., 2008). This is a rare genetic disorder that causes brain tumors, seizures, learning disabilities, skin lesions, and kidney tumors. In humans, half of TSC patients are autistic. The results showed that rapamycin was able to reverse mental retardation in TSC mice raising the possibility that this drug may be effective in the treatment of mental disorders associated with autism (Ehninger et al., 2008). In 2010, Galvan and her team published a research showing that rapamycin also improves learning and memory deficits and reduces brain lesions and Aβ levels, in a mouse model of AD, suggesting that rapamycin may have an another exciting use: to fight AD (Spilman et al., 2010). Rapamycin added to diet late in life was also able to extend lifespan in a mice model of aging (Harrison et al., 2009). If these results continue to be repeated and pilot studies demonstrate that treatment works and is safe, rapamycin, which is already approved for other indications, could be utilized—sooner than expected—to prevent behavioral symptoms in autistic children and in AD patients as well as to improve health to the end of life. At this point it will be crucial to understand how rapamycin exerts its positive effect on the brain. It has been suggested that the drug operates in preventing behavioral symptoms in autism and AD and in extending lifespan through a combination of anti-neoplastic effects and effects on cellular stress resistance and response to nutrient dynamics. In a study that has just been published, it has been shown that the levels of three major monoamines (norepinephrine, dopamine and 5-hydroxytryptamine) and their metabolites (3,4-dihydroxyphenylacetic acid, homovanillic acid, and 5-hydroxyindolacetic acid) were significantly increased in midbrain of rapamycin-treated mice compared to controls. The Authors suggest that oral administration of rapamycin, enhances learning and memory in young adults, maintains memory in old C57BL/6J mice, and has concomitant anxiolytic and antidepressant-like effects, possibly by stimulating major monoamine pathways in brain (Halloran et al., 2012). Studies conducted to evaluate the effect of rapamycin in the human coronary artery smooth muscle cell (VSMC) showed that besides its effect as mTOR inhibitor, rapamycin ameliorated imbalance of intracellular cholesterol homeostasis induced by inflammatory cytokines. In particular, in VSMCs, the drug reduced lipid droplets accumulation and CE content induced by IL-1 and increased intracellular cholesterol efflux by upregulating ABCA1 and ABCG1 gene expression. Similar results were also obtained in our laboratories in peripheral cells from AD patients (Pani et al., 2009a; Murai, 2011). When everolimus, the 40-O-(2-hydroxyethyl) derivative of sirolimus, was added to cultured fibroblasts obtained by skin biopsy from AD patients, a significant reduction of CE accumulation was observed (Pani et al., 2009a). In prion-infected N2a cells, Nile red and filipin staining of intracellular lipids reveals changes both in the content and distribution of most intracellular lipids; cell treatments with CE modulators, showed marked anti-prion activity. The most potent anti-prionic effect was obtained with everolimus that drastically inhibited cholesterol esterification. Anti-prion effect also had verapamil, a calcium-blocking drug that inhibits cholesterol trafficking from the PM to the ER, and progesterone, a sterol hormone that affects cholesterol trafficking both from the PM and lysosomes (Orrù et al., 2010c). These results suggest that inhibitors of cholesterol esterification by restoring cholesterol homeostasis may represent a more successful therapeutic approach than drug treatments lowering cholesterol content *per se* (i.e., statins). Notably, our data also point to CE accumulation in peripheral cells as an easy-to-detect hallmark associated with neurodegenerative disorders and/or indicative of increased susceptibility to develop disease.

## Conclusion

In this review we present evidence that preexistent and/or induced modifications of cholesterol homeostasis may create a membrane lipid environment favorable to the initiation/progression of neurodegeneration, and therefore that pharmacologic retrieval of these modifications may represent a successful way to hamper neuronal degeneration. Even if there is still much to be learned about the physiologic function of cholesterol esterification and its precise role in polarized cells such as neurons, CE and ACAT1 appear to be suitable targets for anti-neurodegeneration drug development. In this context, drugs able to reduce the rate of cholesterol esterification, such as ACAT inhibitors, progesterone and rapamycin in particular, are emerging as the best suited for this purpose.

### Conflict of interest statement

The authors declare that the research was conducted in the absence of any commercial or financial relationships that could be construed as a potential conflict of interest.
